# Antioxidant and Cytotoxic Effects of the Methanolic Extract of Eichhornia crassipes Petioles Upon Mg-63 Cell Lines: An In Vitro Study

**DOI:** 10.7759/cureus.38425

**Published:** 2023-05-02

**Authors:** Noufal K P, Rajesh B, Sujith S Nair

**Affiliations:** 1 Department of Anatomy, Bharath Institute of Higher Education and Research, Chennai, IND; 2 Department of Anatomy, Sri Lakshmi Narayana Institute of Medical Sciences, Pondicherry, IND; 3 Department of Pharmaceutics, Crescent College of Pharmaceutical Sciences, Kannur, IND

**Keywords:** osteosarcoma, eichhornia crassipes, dpph assay, cytotoxicity, abts assay

## Abstract

Introduction: *Eichhornia crassipes *(*E. crassipes*) are a longstanding hydrophyte belonging to the Pontederiaceae family and subfamily Trollioideae. It is classified as an invasive plant owing to its phenomenal growth and propagation and is often described as the worst aquatic plant. Natural antioxidants, such as phenolic compounds and flavonoids, have an increased protective effect against free radicals. A single laboratory test is insufficient to comprehend all of the mechanisms entailed in investigating the antioxidant effects of the phytoconstituents. The antioxidant propensity of methanolic extracts from *E. crassipes* petioles was investigated in this study utilizing 2,2'-Azinobis-(3-ethylbenzothiazoline-6-sulfonic acid) (ABTS) and 1,1-diphenyl-2-pycryl-hydrazyl (DPPH). Additionally, the cytotoxic effect of *E. crassipes* methanolic petiole extract upon MG-63 cell lines for the inhibition of osteosarcoma cells was investigated.

Materials and methods: The antioxidant propensity was appraised by employing DPPH and ABTS assays. The cytotoxic effects of the methanolic petiole extract of *E. crassipes *at varying concentrations on MG-63 cell lines were evaluated using the 3-(4,5-dimethylthiazol-2-yl)-2,5-diphenyl-2H-tetrazolium bromide assay. The absorbance scores were computed using the mean and standard deviation. The half-maximal inhibitory concentration (IC50) was calculated by applying probit analysis. The data were analyzed using SPSS Statistics for the descriptive statistics of the percentage of cell viability and regression analysis.

Results: The antioxidant potential was assessed by employing DPPH and ABTS assays at various concentration levels of 50 μg/ml, 100 μg/ml, 200 μg/ml, and 400 μg/ml of methanolic petiole extracts. The antioxidant potential of DPPH (57.95%) and ABTS (60.47%) was more at the elevated doses of 400 μg/mL. The percentage of cell viability upon MG-63 cell line was measured at varying doses of 12.5 μg/ml, 25 μg/ml, 50 μg/ml, 100 μg/ml, and 200 μg/ml of methanolic petiole extracts and was found to be 99.36%, 93.92%, 86.77%, 69.14%, and 45.08%, respectively. The IC50 value for the extract of *E. crassipes* against the MG-63 cell line was 177.65 μg/mL. The regression equation computed from the findings of the probit analysis was y = -0.2881x + 101.18 with a coefficient of determination of R² = 0.992.

Conclusion: The methanolic extracts of the various parts of the plant, such as leaves, flower, rhizome, and petioles, have been established in similar prior studies to contain the highest phenolic constituents and were found to have a high rate of DPPH radical scavenging activity and reducing power. It is inferred from the findings of the present study that *E. crassipes* petiole extracts have a significant protective role against oxidative stress, potentially attributed to the antioxidant potential. Further, the findings of the study reveal that the methanolic petiole extract of *E. crassipes* induced cytotoxicity upon MG-63 cell lines with an IC50 value of 177.65 μg/mL.

## Introduction

*Eichhornia crassipes* (*E. crassipes*) are a longstanding hydrophyte belonging to the Pontederiaceae family and subfamily Trollioideae [1]. *E. crassipes* is classified as an invasive plant owing to its phenomenal growth and propagation [2]. The usage of herbal drugs for disease treatment has been acknowledged throughout the heritage of ancient cultures. Phytochemicals are significant in the pharmaceutical sector as the sole source of medicine, either as raw materials or as specific drugs. Drugs were administered in their most basic forms as extracts, tinctures, infusions, and herbal remedies that can be applied externally. Water hyacinth is a phytochemical-rich herbal product [1]. The antioxidant capacity of the plant extracts was evaluated frequently using 1,1-diphenyl-2-picryl-hydrazyl (DPPH) and 2,2'-Azinobis-(3-ethylbenzothiazoline-6-sulfonic acid) (ABTS) assays. This DPPH assay evaluates the antioxidant ability of the plant extract to reduce DPPH radicals, either using electron spin resonance identification or by quantifying the drop in absorbance measured by a spectrophotometer. The expression EC50 is commonly used to represent the percentage of antioxidant that induces a reduction of 50% in DPPH absorbance. It is further established that the DPPH and ABTS assays revealed a concentration-dependent antioxidant property of n-hexane, ethyl acetate, and methanol extracts [1,2].

The recognition of antioxidants from vegetable products, specifically polyphenols from plants, has received growing interest as a result of safety and toxicity concerns about synthetic drugs. The most notable natural secondary bioactive metabolites are the phenolic compounds, a class of molecules defined by multi-hydroxyl substitutions to the benzene ring. These chemicals, which include one or more phenolic hydroxyls in their structure, can quickly accommodate free radicals and defend cells from oxidative damage. Thus, *E. crassipes* is a possible alternative to synthetic antioxidants available in the market because of the existence of abundant phenolic content [3].

While water hyacinth is frequently criticized for serious challenges in transportation, agriculture, and energy production, some distinctive features suggest that flora has valuable benefits. Flavonoids, tannins, alkaloids, and phenols are found in the different parts of the plant extracts and exhibit biological properties such as antiviral, antifungal, anticancer, and antibacterial properties. Furthermore, water hyacinth contains a high concentration of oxidative enzymes, non-enzymatic antioxidant mechanisms, tissue healing, and cytotoxic properties. The potent antioxidant characteristics of *E. crassipes* render it a viable and practical reservoir of antioxidants [4]. Numerous studies also prove the occurrence of anti-inflammatory, antiviral, or anti-allergic properties, as well as their protective trait against coronary heart disease, cancer, and other maladies. Flavonoids guard against the effects of ultraviolet radiation, pollution, and dietary additives. Since the human body cannot generate these compounds in a secure manner, they must be acquired from food or other supplements [5].

Reactive oxygen species (ROS) were linked to the pathogenesis of a wide range of ailments, notably neurodegenerative disorders, malignancies, mild cognitive deficits, coronary heart disease, Alzheimer's disease, atherosclerosis, ulcerative colitis, cataracts, and inﬂammatory reactions [6]. Osteosarcoma is the leading type of metastatic bone cancer, accounting for around one-third of all bone cancer cases. It occurs most commonly in the metaphyses of long bones in young individuals exhibiting greater potential for growth [7]. Flavonoids and phenolic compounds have indeed been identified to be effectual antioxidant agents. Various synthetic antioxidants are currently being used. However, it has been observed that they have various negative effects, including the threat of liver failure and cancer in laboratory animals. As a result, more efficacious, least toxic, and cost-efficient antioxidants are warranted. Medicinal plants tend to possess these desirable comparative benefits, which explains the increased interest in antioxidants derived from plants [6].

Several drugs with anti-inflammatory, anticarcinogenic, and hepatoprotective properties have often been noticed to have an antioxidant or neutralizing free radical action as a component of the biosynthetic pathway. The generation of ROS from active neutrophils and macrophages is hypothesized to have a role in the inflammatory response process. This overproduction causes cellular injury by degrading macromolecules and causing membrane lipid peroxidation. Furthermore, ROS fosters inflammation by encouraging the production of cytokines which increase the infiltration of more macrophages and neutrophils. As a result, hydroxyl radicals are essential agents that initiate or perpetuate inflammatory pathways. They are neutralized by the antioxidant propensity of phytoconstituents which in turn assist in reducing inflammation [8].

Natural antioxidants, such as phenolic compounds, carotenoids, and flavonoids, have a high biological value and, thus, have an increased protective effect against ROS and free radicals [9]. To the finest of our knowledge, studies on the antioxidant properties of *E. crassipes* petioles are scarce. A single laboratory test is insufficient to comprehend all of the mechanisms entailed in investigating the antioxidant effects of the phytoconstituents [10]. While plants generate antioxidants to combat the oxidative stress induced by sunshine and oxygen in the atmosphere, they have become a reservoir of important novel antioxidant molecules. The commercialization of plants as antioxidant providers to improve both nutrition and food preservation is of current relevance. Research studies have demonstrated evidence of an association between the intake of phenolic-rich substances in foods and beverages and the lowering of the risk of illness. These benefits have been related to antioxidants found in plants such as flavonoids, folic acid, tocopherols, and carotenoids. Spectroscopy of the segregated fractions indicated the existence of many chemicals that served synergistically in the crude extract, resulting in its highest activity [9,10]. Henceforth, the antioxidant propensity of methanolic extracts from *E. crassipes* petioles was investigated in this study using ABTS and DPPH. Additionally, the cytotoxic effects of *E. crassipes* methanolic petiole extract upon MG-63 cell lines for the selective inhibition of osteosarcoma cells were investigated.

## Materials and methods

Extraction of the plant materials

*E. crassipes* were gathered from the water bodies of Ezhikkara, Ernakulam district, Kerala, India. A competent botanist authenticated the plant species. The petioles of the obtained plant were collected and shade-dried. The samples were chopped into tiny fragments and immersed in 70% methanol for seven days at room temperature. The extract was then subjected to decantation, vacuum-filtration, and concentrated in a rotary evaporator followed by lyophilization. The powdered extract was employed in a Soxhlet extractor which was programmed to run 10 to 15 cycles [5,9,11].

In vitro appraisal of cytotoxic effects by MTT assay

The methanolic petiole extract was considered for the 3-(4,5-dimethylthiazol-2-yl)-2,5-diphenyltetrazolium bromide (MTT) assay in accordance with the prior work of phytochemistry [11] since it yielded a greater concentration of cytotoxic constituents. MG-63 osteosarcoma cell lines were purchased from the National Centre for Cell Sciences, Pune, India. The sample size was estimated to be 5000 cells/well based on a prior similar study on the cytotoxic effects initiated by Abel and Baird [12].

Culture media, its maintenance, and cell preservation

Dulbecco's Modified Eagles Medium (DMEM, Himedia, USA) was used to culture the cells, which was reinforced with 10% fetal bovine serum (FBS) and a 1% antibiotic mixture comprising 100U/ml of penicillin, 100g/ml of streptomycin, and 2.5g/ml of amphotericin B. Cell-containing 25 cm2 tissue culture (TC) flasks were incubated at room temperature, 5% carbon dioxide, and humidity. The cells were stored in customized cell culture media augmented with 20% FBS and 10% dimethyl sulfoxide (DMSO) or glycerol at reduced passage quantity in the cryogenic nitrogen vapor. A mixture of 0.025% trypsin and 0.01% ethylenediaminetetraacetic acid in phosphate-buffered saline was applied to a thin film of cell cultures in TC flasks. Trypsinized cell lines were diluted in the culture medium to a concentration of 5 x 103 cells per well in 100 µl. Cells were implanted into 96-well plates and incubated for three to four days [11].

Sample preparation, treatment, and microscopic observation

The test sample was treated in 100 mg/ml of DMEM media and filtered using a 0.2 µm membrane filter. The Sample was then diluted further in DMEM media and implanted in the 96-wells plated cells cultured at final doses of 12.5 µg/ml, 25 µg/ml, 50 µg/ml, 100 µg/ml, and 200 µg/ml respectively. Untreated wells were used as control. The average of triplicate measurements was employed to reduce errors. The treated and the control wells were captured using the MICAPS HD camera at fixed intervals for 24 hours in the Labomed TCM-400 inverted phase contrast microscope. Cytotoxicity was marked as any perceptible alterations to the morphological features of the cell.

The media was extracted from the cell culture wells and disposed after 24 hours following incubation. The wells were filled with 100 µl of 0.5 mg/ml MTT solution in DMEM media. The plates were then left to incubate for another two to four hours to enable formazan crystals to develop. The supernatant was eliminated, and 100 µL of 100% DMSO was incorporated into each well. A microplate reader was used to determine the absorbance at 570 nm. The two wells of each plate were left empty to function as controls. The average of triplicate values was computed. The percentage of cell inhibition was estimated using the following equation:

% of viable cells = (Average absorbance of treated sample/Average absorbance of control sample) x100

The high-quality reagents and compounds, such as ABTS, DPPH, and potassium persulfate, were purchased from Merck, Mumbai, India.

ABTS radical scavenging assay

This method was modified from that stated by Re et al. [13]. The ABTS solution of 7 mM was mixed with a 2.45 mM of potassium persulfate solution and left in the darkness for 16 hours to form a solution of ABTS radical cation. The radical cation was then dissolved with 50% methanol before use in the test, yielding an initial absorbance of approximately 0.70±0.02 at 745 nm. In a microcurette, 3 ml of ABTS working reference was blended with 300 μl of the test specimen to determine free radical scavenging capability. After six minutes, the absorbance reading was obtained. The percent of inhibition was estimated using the formula: % of cell inhibition = (Abs745 control - Abs745 test sample / Abs745 control) x 100.

DPPH radical scavenging assay

The DPPH radical-scavenging action of sequential extracts of *E. crassipes* was determined by employing Chang et al.'s technique [14]. In summary, 10 µl of test specimens were combined with 90 µl of 50 mM Tris-hydrochloric acid buffer with a pH of 7.4 and 200 µl of 0.1 mM DPPH-ethanol solution. The diminution of the DPPH radical was evaluated following incubation for 30 minutes at room temperature by monitoring the absorbance at 517 nm. DPPH served as a positive control. The test sample was repeated thrice. The percentage of cell inhibition was computed using the following equation:

% of inhibition = (Abs517 control - Abs517 sample / Abs517 control) x 100

Statistical analysis

The data were analyzed using SPSS Statistics Version 26.0 (IBM SPSS Statistics for Windows, Version 26.0, Armonk, NY) at a 5% significance level (p<0.05). The normality of the data was verified by applying the Kolmogorov-Smirnov test. The absorbance measurements were used to assess the proportion of cell inhibition (mean±SD). The half-maximal inhibitory concentration (IC50) was computed using the probit model and the slope of the regression formula, y = mx + c.

## Results

The free radical scavenging propensity of the methanol extract of *E. crassipes* petioles was appraised by the DPPH and ABTS assays. The average of triplicate measurements was considered in the study (Table 1).

**Table 1:** Antioxidant activities of the methanolic extracts from E. crassipes petioles in DPPH and ABTS scavenging assays ABTS: 2,2′-Azinobis-(3-ethylbenzothiazoline-6-sulfonic acid) DPPH: 1,1-diphenyl-2-pycryl-hydrazyl

The volume of the sample (µg/mL)	% of DPPH scavenging mean ±SD	% of ABTS scavenging mean ±SD
50	11.16±0.74	14.62±1.33
100	20.57±0.87	30.01±0.84
200	38.63±0.94	46.73±1.17
400	57.95±1.26	60.47±1.20
Control OD	0.48	0.30

The scavenging action was in line with the concentration of the extract, implying that it was an excellent source of naturally occurring antioxidants. The antioxidant potential was assessed at various doses of 50 μg/ml, 100 μg/ml, 200 μg/ml, and 400 μg/ml of methanolic petiole extracts. The least percentage of DPPH and ABTS scavenging activity was found to be 11.16±0.74 and 14.62±1.33, respectively, at 50 μg/ml of the extract. At an elevated dose of 400 μg/mL, the methanol extract showed the highest percentage of antioxidant action of DPPH (57.95%) and ABTS (60.47%).

Table 2 illustrates the findings of the probit analysis of the methanol extract of *E. crassipes* petioles.

**Table 2:** Probit analysis of the methanolic petiole extract of Eichhornia crassipes against MG-63 cell lines ABTS: 2,2′-Azinobis-(3-ethylbenzothiazoline-6-sulfonic acid) DPPH: 1,1-diphenyl-2-pycryl-hydrazyl

			Drug concentration unit: μg/ml (cell line: MG-63)				
Parameter	Blank	Untreated	12.5 µg/ml	25 µg/ml	50 µg/ml	100 µg/ml	200 µg/ml
Abs reading 1	0.04	0.88	0.861	0.83	0.75	0.64	0.43
Abs reading 2	0.05	0.89	0.869	0.82	0.76	0.62	0.40
Abs reading 3	0.04	0.86	0.884	0.84	0.78	0.60	0.43
Mean abs	0.04	0.88	0.87	0.83	0.77	0.62	0.42
Mean abs (sample blank)		0.83	0.83	0.78	0.72	0.58	0.38
SD		0.01	0.01	0.01	0.02	0.02	0.01
% cell viability		100	99.36	93.92	86.77	69.14	45.08

**Figure 1:**
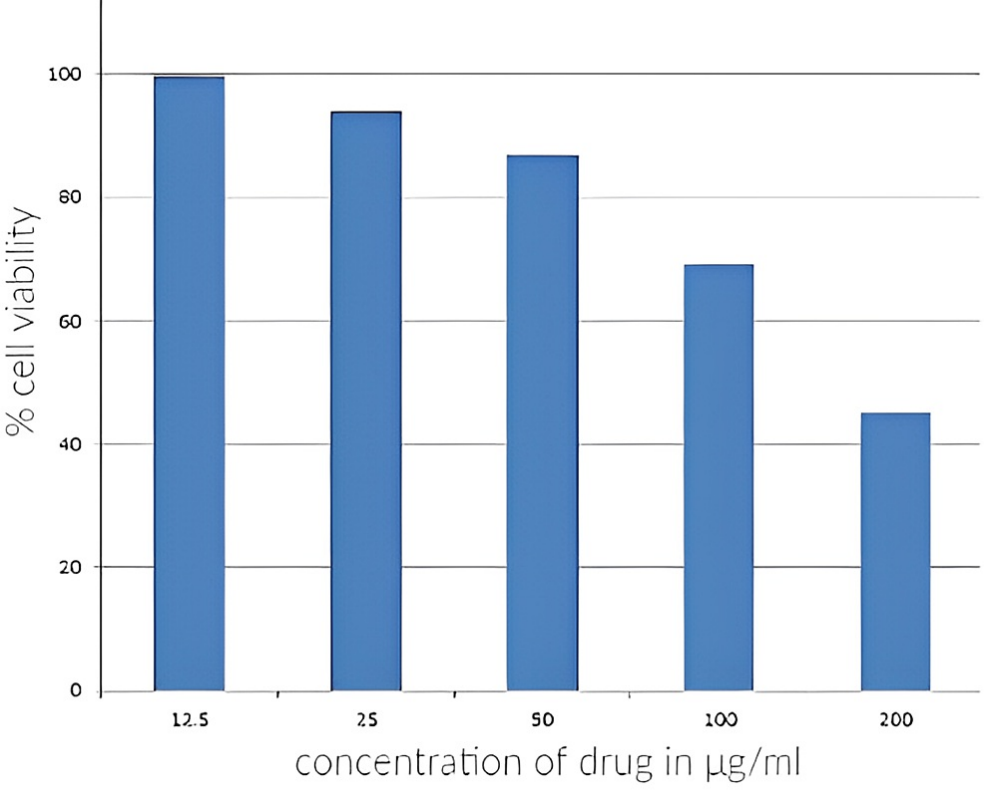
Cytotoxic action of the methanolic petiole extract of E. crassipes against MG-63 cell lines

The cytotoxic effects of the methanol extract of *E. crassipes* petioles against MG-63 cell lines revealed a decreased viability of the cells at higher doses (Figure 1).

**Figure 2:**
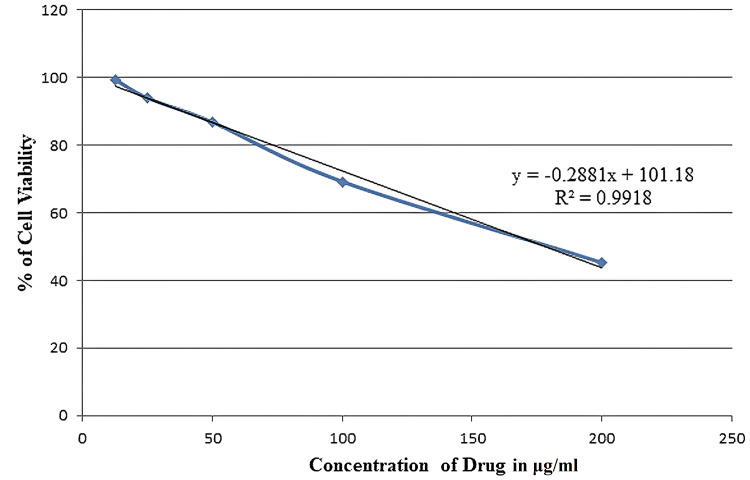
Dose-response relationship of the methanolic extract of E. crassipes petioles against MG-63 cell lines

The proportion of cell viability was measured at concentrations of 12.5 μg/ml, 25 μg/ml, 50 μg/ml, 100 μg/ml, and 200 μg/ml of methanolic petiole extracts and was found to be 99.36%, 93.92%, 86.77%, 69.14%, and 45.08%, respectively. The IC50 value for the extract of *E. crassipes* against the MG-63 cell line was 177.65 μg/mL. The regression equation computed from the findings of the probit analysis was y = -0.2881x + 101.18 with a coefficient of determination of R² = 0.992 (Figure 2).

## Discussion

The current study found that *E. crassipes* had antioxidant properties and cytotoxic effects of *E. crassipes* against MG-63 osteosarcoma cell lines in vitro. Phytochemicals have been shown to interact with a variety of molecular domains such as transmembrane transporters, hormones, enzymes, and receptors of neurotransmission. As a result, a rising number of new vegetation types and by-products are indeed being found and examined for possible application in the pharmaceutical, medicinal, and related industries [15]. Numerous in vitro studies have already documented the antiproliferative potential of the various extracts of different parts of the *E. crassipes* against various types of cancers [10,15,16]. However, to the best of our knowledge, the present study is the first of its kind to establish the cytotoxic effect of the methanol extract of *E. crassipes* petioles against MG-63 cell lines. Cysteine-based proteins, such as metallothionein, have been found in *E. crassipes*. Cysteine is a vital ingredient of glutathione and has antioxidant properties against oxygen and hydroxyl free radicals [16,17]. ROS encompass hypochlorite radical, hydrogen peroxide, hydroxyl radical, singlet oxygen, nitric oxide radical, and lipid peroxides [18]. Phenolic compounds serve as donors of hydrogen and quenchers of singlet oxygen attributable to the redox characteristics [19].

The antioxidant potential of extracts produced from *E. crassipes* petioles was investigated in two principal in vitro methods in the current study. These findings were consistent with prior research that proved the antioxidant property of phytochemicals [6,7,16]. The biochemical assays determine the widely available and reliable antioxidant potential of phytoconstituents. Because different antioxidants produce different responses in different testing systems, it is critical to employ a variety of antioxidant tests to comprehend the role of the active constituents. The removal of free radicals by antioxidants entails the exchange of hydrogen for a free radical. As a result, the radical reactivity is caused by reducing it to an unreactive species by eliminating the odd electrons trait. Because of their phenolic composition, plants serve as electron donors which is attributable to the DPPH radical scavenging action as interpreted by the findings of the present study. This finding was concurrent with the previous research revealing that the DPPH scavenging abilities of phytoconstituents are enhanced with higher doses [20]. Various antioxidant assays were generally compatible with each other as indicated by Kasangana et al. [21].

The ABTS method is a well-known, convenient approach for determining antioxidant effects and could be a valuable tool for evaluating samples to acquire naturally occurring high levels of antioxidants in foodstuffs [20]. The coupling is a characteristic antioxidant response of phenolic compounds wherein the coupling adducts with ABTS+. Furthermore, oxidative breakdown of these coupling adducts results in hydrazindyilidene-like and/or imine-like adducts with 3-ethyl-2-oxo-1,3-benzothiazoline-6-sulfonate and 3-ethyl-2-imino-1,3-benzothiazoline-6-sulfonate as biomarker chemicals, respectively. The coupling process affecting the total antioxidant propensity, its specificity, and the importance of antioxidant constituents needs to be investigated further [22].

The scavenging action of DPPH at 517 nm is frequently utilized to assess the antioxidant effects of naturally produced foods and plants [20]. The antioxidant combines with DPPH to form 1,1-diphenyl-2-picryl hydrazine. DPPH is deemed to be a suitable peroxyl reactive kinetic model, characterized by the dissociation of the excess electrons. The addition of H+ ions to the DPPH radicals causes a shift in color from purple to pale yellow [7]. The drop in absorbance at 517 nm demonstrates the capability to remove DPPH radical scavenging activity [23]. We also assume the phenolic compounds which have been identified in the extract of *E. crassipes* contribute significantly to the reported cytotoxic activity. Previous studies have demonstrated that the phenolic compounds have antitumorigenic effects on melanoma, liver, lung, prostate, and ovarian cancer through stimulation of the nuclear factor-kappa B pathway, augmentation of genetic mutation caused by oxidative stress, or inhibition of angiogenesis [11,15,24,25].

Verma et al. demonstrated that the DPPH assay confirmed the existence of antioxidants and documented that the methanol extract outperformed the ethanol extract [26]. ROS are different versions of activated oxygen addressing free radicals including superoxide anion, hydroxyl, non-free peroxyl radicals, and singlet oxygen. These free radicals generally result in proteolysis, lipid peroxidation, and DNA oxidation. They have been associated with several age-related and chronic pathologies [6]. In a related study, Jun et al. noticed the inhibitory effects of curcumin against MG-63 cells on the p-53 signaling cascade. Possible anticancer mechanisms include eliciting the release of oxygen radicals in tumor cells and preventing the G1 phase of the cell cycle through the activation of apoptotic cell death [27].

Oxidative stress is distinguished by the presence of metabolites known as free radicals. ROS are produced under normal physiological settings but then become harmful whilst unremoved by endogenous mechanisms. In reality, oxidative stress is caused by a discrepancy between ROS production and antioxidant mechanisms. The in vitro studies and clinical trials have established that ROS are significant repositories of principal mediators that activate oxidation leading to oxidative stress-induced systemic diseases. Abundant ROS is hazardous as it initiates bimolecular oxidation, which causes apoptosis and oxidative stress. Furthermore, oxidative stress induces unintended enzymatic reactivity and oxidative damage to the viable cells [6].

According to Thamaraiselvi et al., the scavenging of free radicals efficiency of 29 µg of ethanolic extracts was identical to the action of 400 µg of ascorbic acid [28]. Tyagi and Agarwal determined the antioxidant potential, total polyphenols, and flavonoids of *E. crassipes* from methanolic extracts of various sections of plants and demonstrated their therapeutic and pharmaceutical values [29]. According to Nugriani et al., the powdered preparation of *E. crassipes* displayed higher TPC and antioxidant properties with a proportion of 1:2 than the various liquid extracts [30].

The study limitation is that it is an in vitro study; hence, clinical applications have not been done, and the sample antioxidant capabilities are tested with fewer samples.

## Conclusions

The study revealed a novel finding that the methanolic petiole extract of *E. crassipes* induced a cytotoxic effect upon MG-63 cell lines with an IC50 value of 177.65 μg/mL when varying doses of concentrations from 12.5 μg/mL through 200 μg/mL were investigated. It is further inferred that the antioxidant characteristic of DPPH and ABTS assays increased with increasing concentrations of active ingredients. At 400 μg/ml of extract, approximately 60% radical scavenging or antioxidant action was accomplished. *E. crassipes* petiole extracts have a significant protective role against oxidative stress, potentially attributed to the antioxidant potential. Thus, the present study serves as a wake-up call and offers valuable data for future optimization of polyphenolic extraction from *E. crassipes*.
